# Cases of Obscure Pneumonia in Children, with Remarks

**Published:** 1851-01

**Authors:** J. Forsyth Meigs

**Affiliations:** Philadelphia


### MEDICAL EXAMINER,

AND

### RECORD OF MEDICAL SCIENCE.

NEW SERIES.—NO. LXXIII.—JANUARY, 1851.

---

### ORIGINAL COMMUNICATIONS.

---

### *Cases of Obscure Pneumonia in Children, with Remarks.* By J. Forsyth Meigs, M. D., Philadelphia.

(Read before the College of Physicians, Philadelphia, on Tuesday, Nov. 5th, 1850.)

I desire to call the attention of the College to a few cases illustrative of the difficulties which sometimes occur in the diagnosis of pneumonia in children. Inflammation of the lung is very easily recognized in early life, when it assumes, as it generally does, its frank and open form. When the rational symptoms of the disease, the cough, pain in the chest, rapid breathing, high fever and great thirst, are present in a marked degree, there is no difficulty in understanding that the patient is laboring under a pulmonary inflammation. The greater dryness of the cough in the beginning, the greater intensity of the pain, the higher grade of the fever, and the smaller amount of wheezing, as heard independently of auscultation, will almost always allow us to distinguish even between pneumonia and bronchitis, without a resort to the physical means of diagnosis. But when, on the contrary, the local symptoms are slightly marked, or almost null, and when, from some peculiarity of the individual, the chief symptoms in the case are such as would seem to indicate disease of some other system of the body, as the nervous or digestive, the diagnosis of the pulmonary inflammation becomes exceedingly difficult, and to one who either neglects or is unable to avail himself of the benefits of auscultation and percussion, impracticable. So obscure, indeed, in some instances, are the rational signs of pneumonia in children, and so prominent are those appearing to indicate disease of some other part of the body, that the attention is drawn away almost irresistibly from the real seat of mischief, and the true nature of the illness is misunderstood, until, from the further development of the case, the pulmonary symptoms grow into greater significance, or from the very darkness which hangs over the diagnosis, the physician resorts to a thorough physical examination, and so detects the existence of the pneumonia.

Now the causes which tend to embarrass the diagnosis of this inflammation in children may be referred, it seems to me, to two principal conditions: first, that in children the nervous system is so much more susceptible to the action of disturbing agents, and therefore so much more apt to suffer derangement of its functions in all states of disordered health, that the symptoms issuing out of this circumstance assume at that period of life much greater severity and prominence than at later periods. Second, that in children the physical signs of thoracic disease are much more obscure and uncertain than in adults, because of certain physiological and pathological differences which characterize the former age, and because of the obstacles presented by the general unwillingness, or even refusal of the child, unless constrained, to submit to the examination.

That the nervous system is especially subject to derangement in children is so trite an observation in medicine, that it scarcely needs any comment on my part. Nevertheless, I will recall to your memories the great frequency of spasmodic and convulsive disorders, and of organic diseases of the nervous system in early life. How frequently, moreover, do we see children presenting the most serious, nay, the most dangerous nervous symptoms, in disorders which in adult patients would be regarded as trivial, or at least entirely destitute of danger. How common to meet with the most violent fever, with alarming jactitation, with irregular and tremulous muscular movements, with delirium, drowsiness, stupor, or coma, with terrifying convulsions, and even with fatal congestion of the brain or apoplexy, in cases of simple indigestion or of intestinal disorders, in the fever depending on simple angina, and at the onset or in the course of the different fevers and phlegmasiæ. These things happen daily. They are common in the experience of every man conversant with the diseases of infancy and childhood. Need we be surprised, therefore, to find this class of symptoms occurring frequently at the beginning, in the course, or at the termination of pneumonia, one of the most severe and dangerous inflammations to which the constitution is subject? And is it not easily to be understood, also, that, when such symptoms make their appearance in a marked form in the pneumonia of children, and especially when they occur at the onset of the disease, they must more or less confuse and embarrass the diagnosis. How can it be otherwise? A sick child has violent fever, drowsiness, stupor or delirium, sensibility to light, headache, vomiting, perhaps constipation; when partially aroused he is irritable and his movements are tremulous: he has few or none of the rational signs of pneumonia; little or no cough; no complaints of pain in the chest; on the contrary, he complains only of pain in the head; his respiration is more frequent than natural, but less so than in ordinary frank pneumonia;—how, I will ask, is one to escape from the idea that a child presenting these symptoms is laboring under some inflammation of the brain? It is most certainly an embarrassing and delicate point to judge upon.

But the diagnosis of pneumonia is rendered obscure and difficult in children, not only by the greater irregularities observed in the rational symptoms, but also by certain conditions which make the application of physical exploration in subjects of a tender age less useful than in those who have reached the period of maturity. Amongst these conditions the most prominent are the following: The thorax is naturally so much more resonant in early life as to afford a much less certain indication, by dulness on percussion, of the presence in the thoracic cavity of conditions diminishing the amount of inspired air; or, in other words, the healthful sonoriety of the chest is so great as to permit of a certain degree of induration of the tissues of the lung, which yet shall not be appreciable to an ear of ordinary acuteness or cultivation, because there still remains an amount of sound on percussion sufficient to deceive the great majority of observers.

A second cause of difficulty, connected, like the last, with the physiological construction of the thorax, is the fact that the respiratory murmur in the child is much louder and stronger than in the adult; it is, in fact, puerile; it has somewhat of a blowing or bronchial sound, and therefore renders the detection of bronchial respiration, one of the most important signs of pneumonia, much less easy and positive than during the maturer periods of life.

In addition to these two causes connected with the physiological conditions of childhood, we have certain pathological states to increase the embarrassment under which we labor. The most important of these is, that pneumonia is so often double, as to make us lose very frequently the advantage of the comparison of the two sides; and unless care is observed, the physician is led into error, by falsely concluding from the fact of the two sides being equally sonorous, that the sound he elicits is natural; whereas, in truth, both sides are considerably duller than they ought to be. Again, from some cause, crepitant râle is much less to be depended upon in the child than in the adult; either because it is really absent, or because the ear is unable to detect it, owing to the child's refusing or being unable to distend the thorax sufficiently to cause the air to enter a sufficient number of the air-cells to produce this particular morbid sound.

Last, but not least, our means of diagnosis are still further restricted by the opposition which the child so often makes to the examination. Who has not been annoyed and tormented, and even foiled, when the case is an obscure one, and therefore requiring great delicacy of perception, by the patient's resisting with cries, and screams, and struggles, every attempt at a proper exploration? Or, if not foiled, how much more uncertain and unsatisfactory are the results obtained under these circumstances, than they are in the instance of adult patients.

After these prefatory remarks upon the subject to which I have requested your attention, I shall proceed to relate the history of three cases that occurred in my own practice—cases which I hope will not weary, by their detail, the patience of the College, since they are strongly illustrative of the perplexing symptoms which sometimes accompany, and which tend to mask the pneumonia of children.

The first case is one in which the onset of the disease was so much like that of meningeal inflammation, as to render the diagnosis very difficult in its early stage. It is as follows: A fine, hearty boy, between six and seven years of age, born of very healthy parents, both of whom were living, had an attack of anginose scarlatina, of moderate severity, in the month of April. He recovered from this sickness without any difficulty, and resumed his attendance at school. On Wednesday, the 15th of May, he was seized with the symptoms of cold in the head, and had some fever, and a severe earache. On Thursday he was much better. On Friday he was rather imprudently taken out to ride, the weather being raw and disagreeable, and he consequently fell sick again on the evening of that day. The next day, Saturday, he had violent fever, much pain in both ears, severe frontal headache, and great sensibility to light when exposed to it. He was at the same time very drowsy, sleeping nearly the whole day; but could be roused when loudly and vehemently spoken to, so as to answer a few questions, and manifest great irritability. There was, in connection with these symptoms, frequent vomiting. The bowels had not been moved. There was no cough whatever. The respiration was slightly quickened. I suspected an attack of pneumonia, but could not detect it upon the most careful examination. I prescribed for him two grains of calomel in a dessert spoonful of castor oil, and a strong mustard foot bath.

On Sunday (2d day), the symptoms were much the same. The headache was very severe, and the only thing complained of. The sensibility to light continued excessive. There was frequent vomiting, the smallest quantity of water even, being generally rejected at once. The bowels had been freely moved. There was no full cough, but he had given a short hack two or three times. The respiration was decidedly accelerated, rising to 35 or 40. The skin was hot and dry, and the pulse 130 in the minute, and at the same time rather hard, but perfectly regular. I still suspected pneumonia, but could not help feeling uneasy in regard to the brain, particularly as the most careful exploration failed to reveal any signs of pneumonia. I ordered iced lemonade and sweet spts. of nitre as the remedy, with cloths wet with cold water to the head, a mustard foot-bath twice a day, and a mustard poultice to the epigastrium.

Monday, (3d day.) No change, except that the respiration has risen to 45 in the minute, unattended, however, by any labor or difficulty whatever. He has coughed three or four times again. The cough is short and dry, and not painful. The pulse is 132, jerking. The vomiting continues with most distressing retching. The tongue is clean, moist, and disposed to be reddish. Auscultation to-day reveals well marked bronchial respiration before and behind, over the upper lobe of the right lung, but no râle whatever. Over the same region the percussion was dull. The prescription was leeching to four ounces over the right scapula; acetum opii, one drop every two hours; a mustard poultice over the front of the thorax, and a mustard foot-bath.

Tuesday, (4th day.) Better. Less fever. Less headache. Eyes not so sensitive to light. Vomiting has moderated, though it still recurs from time to time. To take the black drop occasionally.

Thursday, (6th day.) The fever has disappeared. The cough is much more frequent than before, and loose. Percussion over inflamed point less dull. The bronchial respiration is being replaced by vesicular murmur. Black drop at night; diet to consist of milk and bread, ice cream, etc.

Saturday, (8th day.) The patient is convalescent. He coughs but little, not more than half a dozen times a day, and then loosely. There is no fever. No vomiting for the last two days. Head clear. Tongue red, clean, moist. No appetite. No pain of any kind. Sleeps well and takes no medicine. The dulness on percussion is diminishing, though still very clearly marked. Bronchial respiration much less distinct. To-day, for the first time, I can hear, just at the end of a long inspiration, which he now voluntarily takes for me, a very fine, but distinctly perceptible crepitant râle.

From this time he rapidly and entirely recovered.

*Remarks.*—This case appears to me a very interesting one from the obscurity which attended it at the onset. A healthy boy, between six and seven years old, who has had a slight cold in the head, with earache, but who has apparently recovered, is taken out to ride. On the evening of that day he falls sick again, and on the following day when I see him, I find him suffering with severe fever, violent headache and pain in both ears. He is at the same time exceedingly drowsy and inattentive, and when partially roused from this condition, complains bitterly of the light, and is very cross and irritable. He has no cough whatever, and, beyond slightly quickened breathing, no symptom of thoracic inflammation. He has frequent vomiting, rejecting nearly every thing that he takes, even to water. His bowels had not been moved within a day.

Now what was the nature of this attack? Was it meningitis? Scarcely tubercular meningitis, since the attack was so sudden, and the patient healthy, and descended of vigorous parents. Was it acute simple meningitis? This was more probable, especially if we adopt the idea of an extension of inflammation from the structures of the ear to the coverings of the brain. But there was no delirium whatever, which there would probably have been had meningeal inflammation gone far enough to have produced the drowsiness and sensibility which the child evinced. Moreover, the pulse was not over 130, and the skin not intensely hot, which would almost certainly have been the case had the attack been one of the kind I am supposing. Add to these considerations, those of the child's having had a cold, and just as he was recovering, having been exposed during a ride to a raw May wind. Explain his vomiting by the circumstance that he and all his brothers, of which there were several, inherited from their mother a strong disposition to that symptom in all their attacks of sickness, and I think there were reasons enough for hoping that the nervous symptoms, under which he labored, and which, for the time being, were the only ones he presented, depended not upon cerebral disease, but were sympathetic merely with some local inflammation of another and distant organ, and what organ so likely to be the seat of the trouble, as the lung?

On the next day, when I heard that he had coughed several times, that the bowels had readily responded to medicine, and that his nervous symptoms had made no progress, which they ought to have done, had they depended on inflammation of the meninges, I felt still more convinced that the case must be one of pneumonia, though I could not as yet detect any signs of that disease by the most careful physical exploration. On the third day, however, the nature of the attack became clear and decided, for I detected dulness on percussion and bronchial respiration over the upper lobe of the right lung. There was no longer any doubt or obscurity, though the stupor, sensibility to light and vomiting, remained unabated.

I shall next detail the history of a case intended to show how serious and how conspicuous the nervous symptoms sometimes become in the latter stages of pneumonia. I select this case because it affords a very strong example of the extreme gravity which this class of symptoms occasionally presents at that period of the disease, and because also it shows how mistaken is the notion that severe cerebral symptoms occurring in the course of various local inflammations depend generally upon a transfer of diseased action to the brain: whereas, in fact, they are almost always merely sympathetic phenomena; or still more frequently, are occasioned by that state of the blood which comes at last to be produced by the imperfect performance of the hematosic function in diseases of the respiratory organs, or by the debilitating influence of long sickness, and insufficient supplies of food in diseases of other parts of the body. The case is as follows: On Thursday, the 21st November, I was requested to take charge of a child, who had been ill for seven days under the care of another physician. I was sent for because the original attendant had been obliged to leave town. I was told that the case was one of cold on the chest, in which the disease had left the chest to remove to the brain; that the child was entirely unconscious, excessively prostrated, and, in fact, supposed to be in a dying condition. The parents hoped for nothing from medical treatment, but wished that everything might be done to satisfy their sense of responsibility.

The real history of the case prior to my first visit was this: The patient was a girl between two and three years old, of rather delicate appearance, though seldom sick, and the child of delicate parents, the father having been attacked since his daughter's illness with hæmoptysis followed by many phthisical symptoms. She was taken sick on the 29th of November with slight laryngitis, threatening croup. This was followed by mild catarrh, of which she convalesced on Monday, the 4th of December, under the care of the family physician. She remained well now until Tuesday, December the 12th, when she was again seized with cough and fever, and the physician was again sent for. This gentleman now attended her daily until Saturday the 16th, when he thought her better. During these five days she had cough, quick breathing and fever, and on Thursday morning some threatening brain symptoms, for which a blister was applied to the nucha. On Sunday, the sixth day of her sickness, she was dull and indifferent. On Monday she scarcely recognized her mother, and on Tuesday not at all. During all this time, and up to Thursday, Dec. 21st, the tenth day of the sickness, when I first saw the child, she had had no spontaneous vomiting, but had vomited several times after taking her medicine, which consisted of a calomel and tartar emetic mixture. She was not constipated, but had regular daily evacuations. The treatment had consisted at first in the use of a calomel and tartar emetic mixture, and afterwards of blue pill and rhubarb. A blister had been applied to the nucha as already stated.

At my first visit, on the 10th day of the sickness, I found the patient, in truth, in a most alarming condition. My notes give the symptoms as follows: The face is pale and even white, the eyes closed, and the expression dull and stolid. The lips are of a pale red tint, and dry and cracked. The child is very drowsy, indeed almost comatose. She can be roused with great difficulty only enough to glance around languidly and heavily at my watch, for a moment, and then immediately relapses into coma. The eyes are dull and dim, and there are many incrustations about the lids. She neither speaks nor attempts to speak; she recognizes no one, and has neither asked nor made any sign indicative of a wish of any kind. There is no appetite, and no thirst. The pulse is frequent, small, and feeble, and the hands and feet cold, requiring bottles of hot water to maintain any warmth in them. The head and throat are slightly warmer than natural, but not hot. The respiration is 52, short and noiseless, except that a fine wheezing can be heard when the ear is approached near to the chest. There is very little cough—it is short, slight, and rather loose. Auscultation reveals subcrepitant râle over both sides behind. Percussion affords nothing very decided.

I ordered a mixture consisting of six grains of carbonate of ammonia and twelve of quinine in three ounces of vehicle, of which a tea-spoonful was to be given every two hours. A dessert-spoonful of wine whey was to be given every two hours also, and a table-spoonful of chicken tea occasionally. A blister an inch and a half square was applied to the calf of each leg, to be removed at the end of an hour and a half or two hours; and bottles of hot water were kept in contact with the feet, and laid along the sides of the body.

11*th day.*—No very decided change, except that the extremities are somewhat warmer, the pulse a little fuller, and the patient rather more easily roused from her state of insensibility. The remedies to be continued, and a cathartic enema to be administered to open the bowels, which have not been moved.

12*th day.*—Injection operated once last evening. Bowels opened again this morning. Stools natural. Temperature good. Rather more color in the face. Patient more intelligent, and much more easy to be roused; observes her mother and recognizes her for the first time, but makes no attempt to speak. She is very weak, trembling very much on the least attempt at motion. Pulse fuller and stronger. Respiration easy. Takes her food readily. Continue treatment.

13*th day.*—Decidedly better. Is now wide awake, with a natural and intelligent expression of countenance. Face much less pale; lips well colored, and less dry: eyes less dull; evidently recognizes those around her, though she does not attempt to speak as yet. Whole surface warm and dryish, and the hands rather hot indeed. Pulse 120, fuller, and stronger, though still small. Respiration 60, not quite regular, short but without any labour. Cough much more frequent than before and stronger. It occurs sometimes in spells, and is disposed to be loose, though still rather hard. No vomiting. Bowels opened twice since last night; the dejection being natural, but rather free. Takes her food very well. Not very thirsty. The abdomen is somewhat full, but soft and supple; some tympany. The patient picks her nose and lips a great deal, and on the 9th and 10th days picked the skin over the neck and upper part of the sternum so much as to produce angry looking pimples and excoriations. Auscultation reveals to-day vesicular murmur, rather feeble, over the front of the thorax, vesicular murmur over the upper part of the chest behind, and fine subcrepitant râle with bronchial respiration both in inspiration and expiration, over the lower half of both sides behind. Percussion is somewhat dull over the lower part of the dorsal region, and is rather less clear on the right than on the left side.

The carb. amm. and quinine mixture is to be suspended, and a teaspoonful of a mixture consisting of four grains of iodide of potassium in half an ounce of sarsaparilla syrup, and an ounce and a half of water, is to be given every three hours; wine whey to be continued. Diet to consist of chicken tea, and milk and water. One grain of Dover's powder, to be administered every evening.

On the 14th day the child cried loudly and obstinately for the first time, and for the first time in many days, shed tears. The pulse was 124, of good volume, soft, and regular. The breathing was 70, high, regular, and without effort. Skin warmer than natural, and dry; tongue moist, and covered with dark brown fur.

On the 16th day, I found her slowly improving in strength. She now remains awake the greater part of the day, and is much amused with her toys; the appetite is moderate, and the thirst not too great; the cough is more frequent, occurs in spells, and is rather hard. Has had no natural stool to-day; the pulse is between 120 and 130, rather small and feeble; the breathing is 50, still high, but not labored. The respiratory sound is feeble, and attended with some expiratory blowing, and a fine rare subcrepitant râle over the lower half of both lungs behind; percussion over same regions is slightly duller than natural, and a little more so on the right than on the left side. Tongue clean and moist; lips dryish, and much picked at by the child; abdomen natural; soft. Sleeps well at night with the Dover's powder.

The mixture of Iod. potass. to be continued; milk punch to be be given in the dose of a tablespoonful every three hours. Ice cream, rice gruel, and a little chicken for diet. The grain of Dover's powder to be given at night.

From this time the patient continued to go on very favourably in every respect, so that on the 21st day of the illness, the pulse had fallen to 95, and the respiration to 35; whilst the strength was rapidly improving, and the cough had become much less frequent and looser. The bowels were regular; there was no diar. rhœa. The appetite was increasing, the thirst had disappeared, and the spirits had become much more cheerful and pleasant.

The Iod. potass. was suspended on the 18th day, and a little syrup of ipecacuanha and sweet spirits of nitre substituted; a few drops of brandy were given several times a day in the place of the milk punch.

On the 24th day, the patient was entirely convalescent, as she was gaining flesh, and was able to sit up and amuse herself.

*Remarks.* When I was called to see this case, it was supposed by the family, who were guided by the opinion of the first physician, to be one of disease of the brain, and on that ground was thought to be desperate beyond hope. The opinion of the first practitioner was clearly indicated likewise, by his having applied a blister to the nucha on the third day of the attack, in consequence, it was stated, of some threatening brain symptoms. Upon first seeing the patient indeed, and noticing the extreme stupor, amounting very nearly to perfect coma, I was for a time apprehensive that the case was in reality one of those fatal affections of the great nervous centre, which so rarely yield to the art of the physician. As soon, however, as the history of the case had been fully detailed, I was led to form another opinion in regard to its nature, and one, too, which allowed me to hope that it might yet be turned from the dangerous direction which it had assumed. In fact, the onset of the attack was evidently that of a pulmonary inflammation, and not of cerebral disease. The cough, the quickened respiration, the absence of spontaneous vomiting of marked constipation and of delirium, all looked unlike the approach of meningitis;—and when, in addition to these circumstances, I found that there still remained some glimmering of intelligence, that there had been no spasmodic phenomena, that there was neither dilatation of the pupils, nor irregularity in the direction of the eyes, and that a proper examination of the rational and physical respiratory symptoms, shewed clearly that the child was affected with an extensive catarrho-pneumonia, I had no hesitation in believing that the real disease was inflammation of the bronchia, and of the parenchyma of the lungs, and that the dangerous nervous symptoms which had created so much alarm and even hopelessness, were not primary phenomena, and therefore almost necessarily fatal, but merely secondary or consequential, and for that reason, very much less to be dreaded. Guided by this view of the case, I determined to sustain, and, if possible, to revive the sunken forces of the patient, and by thus gaining time, and restoring to the circulatory and nervous functions, some degree of energy, I hoped that nature might yet be able to carry on the changes necessary in the diseased structures, to restore to them their healthful condition. Effectually, after a few days, I had the great satisfaction to behold my efforts crowned with success. As the pulse became fuller and more active, as the diminished calorific power became invigorated, and as the digestive and absorbing functions grew stronger and more capable of fulfilling their offices, I could perceive that the respiration was augmented in frequency and power, that the cough became more frequent and looser, and that the lungs were gradually and surely relieved of the products of inflammation, which had previously been obstructing and impeding those changes in the blood, which are so essential to the due extrication of the life forces.

I shall now proceed to the history of the last case by which I aim to call the attention of the College to the point I am endeavoring to elucidate; to wit, some of the conditions which so often complicate and obscure the diagnosis of pneumonia in children.

Should I succeed, by my remarks upon this subject, in throwing some light upon a difficult point in practice, or in drawing out from the older and more experienced members of the College, a portion of their accumulated share of experience, I shall not deem the time so boldly occupied by myself this evening, to be wholly misspent.

The subject of the case was a boy between 4 and 5 years of age, of healthy and robust appearance, and born of parents of good, though not vigorous health. This child was seized suddenly, six weeks after an attack of measles, of which he had apparently entirely recovered, with violent fever, great restlessness and distress, vomiting, and distension of the abdomen. When I first saw the patient, these were the only marked symptoms he exhibited. There was nothing to call my attention particularly to the thorax, and I was led to suppose that the fever depended on some gastro-intestinal disorder, from improper eating. A dose of castor oil was administered, to be followed by sweet spirits of nitre, and a warm bath was ordered. On the second day he was much worse. The fever was very high, the skin being hot and dry, and the pulse 160 in the minute by the watch, and jerking. He was now very drowsy and heavy, it being almost impossible to obtain any answers from him, and when obtained they were confused and unintelligible. When disturbed by the examination, his movements were tremulous and uncertain. The tongue was dryish, and very thickly coated. He complained confusedly of pain in the abdomen, which was still distended and sonorous on percussion. There was no sign of respiratory disease, except some quickening of the breathing, and a very slight cough, scarcely to be noticed. I began to suspect that the attack was one of scarlatina, from the extreme quickness of the pulse, which continued at 160 in the minute, from the drowsiness, and from the tremulous, almost spasmodic character, of the muscular movements. Fortunately, however, I was induced, by the obscure character of the symptoms, the increased frequency of the breathing, and the occasional occurrence of a slight cough, to auscultate and percuss the chest. This I was enabled to do only very hastily, and with much difficulty, owing to the boy's unwillingness to submit to the necessary position. I found that the respiration was feeble and obscure over the lower part of the left side behind, and that the percussion was dull over that region. This determined me at once as to the real nature of the sickness. It was pneumonia. I bled him immediately to five ounces from the arm, which I had feared to do before, lest the attack might be one of scarlet fever. Two hours after the bleeding, the pulse had fallen to 140 from 160, which it had been before. The heat of the skin was very considerably diminished soon after the abstraction of blood, and the patient passed a quiet night. The internal remedy consisted of very small doses (30th of a grain) of tartar emetic, with sweet spirits of nitre, every two hours. On the following day, the third, he was much better; the drowsiness and disposition to coma having passed away, the movements become steady, and the heat of skin much reduced. On the fourth day he was out of danger. As the case went on, the signs of pneumonia became unequivocal. The blood drawn on the second day presented a firm, small coagulum, covered with an abundant buffy coat. The recovery took place in the ordinary time, and was perfect.

*Remarks.*—This case was rendered obscure by the facts that the nervous and abdominal symptoms were so severe and prominent as to mask almost entirely the thoracic symptoms. The pulmonary symptoms were indeed not only concealed by the superior and unusual conspicuousness of the nervous and abdominal phenomena, but they were themselves much less marked than in ordinary attacks of pneumonia. It may seem a very easy task to any one who has heard the above case detailed, to have made the diagnosis of pneumonia. But let him who so thinks, imagine a boy between 4 and 5 years old, of nervous and vehement disposition, somewhat spoiled in temper withal, to be taken sick suddenly, with violent fever, pain in the abdomen, flatulent distension of the bowels, so little cough as not to be noticed by the attendants, no evident labor of breathing, heavily coated tongue, and strong disposition to drowsiness. Suppose the child to be still worse on the following day, with a pulse at 160; with stupor, amounting almost to coma; tremulous and uncertain movements; confused complaints of pain in the stomach; distension of the abdomen; a cough, so slight as to be recollected by the attendants only after the strictest examination; no labor of respiration, but merely such an acceleration of its rate as would almost infallibly accompany a pulse of 160: suppose, too, that all this occurs during a season when scarlet fever is prevailing epidemically, and I will ask any one here present, whether the symptoms were not such as might readily lead the mind to suspect an attack either of scarlatina, or, if the abdominal symptoms were chiefly considered, of typhoid fever, rather than one of pneumonia? In fact, I was assisted in the case by a most acute and experienced practitioner, and he was even more disposed than myself, to conclude that the attack was a scarlatinous one, in which the eruption had failed to make its appearance. Fortunately, as has already been said, I was induced, after finding out by minute inquiries of the attendants, that the patient had coughed a few times, to examine the chest; and though the examination was necessarily rapid and imperfect, the result was such as to convince me that the disease was a latent pneumonia. The bleeding and tartar emetic, which were employed as a consequence of this discovery, exerted a most satisfactory influence upon the symptoms, and the child was very soon out of all danger.

I regret, gentlemen, that the details of the case last read to you are so meagre and imperfect, but I give them from my note-book as they were written down under a press of occupation. I can safely, however, make the assertion that they are correct as far as they go; and I have not been deterred, therefore, by the want of greater copiousness of detail, from offering the case to the College as an example of the obscurity which sometimes occurs in the diagnosis of pneumonia in children. I will take this occasion to remark, moreover, even at the risk of wearying your patience, that it has often seemed to me that the dread of not being able to describe a case with all the elaboration and minuteness of finish which is the fashion of the day, has probably prevented not a few of our profession, who are busily engaged in private practice, from attempting a record of the cases which come under their observation; and that the consequence of this may be that many important facts are thus lost to the profession. And yet, surely, a short and even imperfect sketch of a case, written down at the moment of its occurrence, is worth more than a mere recollection, and especially than no record whatever, either memorial or written. If, indeed, a practitioner who has had any considerable share of private business, waits to take notes of his cases, until he can do so with the detail and accuracy of a Louis or an Andral, he would, I suspect, leave but few mementos of his labors. Far be it from me to undervalue the exact and rigorous method of observation insisted upon by the great men of the modern school of medicine,—a method which has given to our science its most useful and durable monuments.

But, let us not, in our admiration of the complete and perfect works of our masters in medicine, contemn the efforts of more modest and humble laborers, since, after all, it is only by slow and patient progression through successive degrees of excellence, that man ever attains that exquisite finish which renders the arts and sciences in their consummated perfection, so attractive to all the finer parts of our nature.

